# Left hemisphere abnormalities in face-selective activation and functional connectivity in developmental prosopagnosia

**DOI:** 10.1162/IMAG.a.971

**Published:** 2025-11-14

**Authors:** Alison Campbell, Xian Li, David Rothlein, Michael Esterman, Joseph DeGutis

**Affiliations:** Boston Attention and Learning Laboratory, VA Boston Healthcare System, Boston, MA, United States; Department of Psychiatry, Boston University Chobanian and Avedisian School of Medicine, Boston, MA, United States; Department of Psychiatry, Harvard Medical School, Boston, MA, United States; Department of Psychological and Brain Sciences, Johns Hopkins University, Baltimore, MD, United States; National Center for PTSD, VA Boston Healthcare System, Boston, MA, United States

**Keywords:** developmental prosopagnosia, occipital face area, fusiform face area, face selectivity, functional connectivity, fMRI

## Abstract

Developmental prosopagnosia (DP) provides a unique opportunity for identifying neural mechanisms that are necessary for normal face recognition, yet the neural basis of DP remains unresolved. We conducted an extensive fMRI investigation of the functional activation and connectivity of ventral and lateral face-selective regions in DP and expanded the analysis to include connections between this face-selective network and all other cortical regions. From a large sample of DPs (N = 34) and controls (N = 23), we found neural differences in DPs in both modalities that implicated the left fusiform face area (FFA). During passive viewing of faces, face-selective activation was reduced in the left FFA and left occipital face area (OFA). During rest, overall functional connectivity within the face network was reduced in DPs, with the largest reductions involving the right anterior superior sulcus (aSTS). Within the face network, DPs showed the greatest reduction in functional connectivity between the right aSTS and the left FFA. However, we found no evidence for functional connectivity differences beyond the areas surrounding the face-selective regions, indicating that functional connectivity deficits in DP are mostly confined to the face network. Overall, these results emphasize the role of the left FFA in face recognition deficits, both in local activation and in its connectivity within the face network.

## 1 Introduction

Human face recognition ability is highly variable, with an estimated 1–3% of the population having such poor face recognition ability that it impairs their recognition of highly familiar faces despite no apparent brain damage (DeGutis et al., 2023; Duchaine & Nakayama, 2005; Shah et al., 2015). This condition, known as developmental prosopagnosia (DP), is highly-selective for faces: recognition accuracy for non-face objects (Fry et al., 2020) and voices (Tsantani & Cook, 2020) is comparable to neurotypical controls (but see Geskin & Behrmann, 2018 for differences in response time). DP, therefore, provides a unique testbed for identifying the neural mechanisms that are necessary for normal face recognition. Early DP research focused on the face-selective occipital face area (OFA) and fusiform face area (FFA) as part of the “core” face network for face perception (Gobbini & Haxby, 2007; Haxby et al., 2000). However, there is still no consensus about how face selectivity is affected in DP. Some have argued that the core face-selective regions respond normally to faces (Avidan & Behrmann, 2021), yet others have reported selectivity deficits both within and outside of the core network (Gerlach et al., 2019; Jiahui et al., 2018; Lohse et al., 2016). Thus, there remains a very fundamental question about whether and where face-selective activation is affected in developmental prosopagnosia.

New behavioral research also indicates that the majority of DPs have either mild (41.3%) or no face perception impairments (31.9%, DeGutis et al., 2024), but large memory-related deficits for the recollection (Burns et al., 2014; Stumps et al., 2020) and associative memory for faces (Palsamudram et al., 2024). These findings raise the possibility that the neural deficit in DP extends to regions beyond those for face perception. Moreover, anatomical and functional evidence has revised the models of face processing, which is now understood to be separated into a ventral pathway that includes the OFA, FFA, and ventral anterior temporal lobe (vATL), and a lateral pathway that includes the posterior and anterior superior temporal sulci (pSTS and the aSTS; Duchaine & Yovel, 2015; Pitcher & Ungerleider, 2021; Y. Wang et al., 2020). Whereas the lateral pathway of the right hemisphere is typically associated with perceiving social interactions (McMahon & Isik, 2023), the left lateral pathway overlaps with regions that have been shown to be critical for semantic processing (Lambon Ralph, 2014), object and face naming (Rice et al., 2018), multimodal identity responses (Tsukiura et al., 2010; Y. Wang et al., 2017), and familiar face processing (Noad et al., 2024). The implication of the lateral pathway for person identity processing (in particular in the left hemisphere) is highly relevant to the identity recognition deficits in DP.

Beyond these face processing pathways, it was also found that functional connectivity between the face network and sensory, social, and memory regions outside of it predicts face recognition ability in neurotypical adults (Ramot et al., 2019). As previous studies of functional connectivity in DP have been mostly restricted to the core network regions (i.e., OFA and FFA; Avidan et al., 2014; Song et al., 2015; Zhao et al., 2018), connectivity of the lateral pathway, as well as connections to regions outside the face network, has yet to be thoroughly investigated in individuals with DP.

In light of these unresolved issues, the goal of the current study was to examine both face selectivity and resting connectivity of the lateral (pSTS, aSTS) and ventral (OFA, FFA, vATL) regions in 34 DPs. This sample is larger than all but one DP fMRI study (Zhao et al., 2018), and the examination of both selectivity and connectivity is rare among DP studies (compared to single modality studies, e.g., Jiahui et al., 2018; Zhao et al., 2018), allowing for a more complete understanding of neural differences in DPs. Further, although studies have often collapsed across hemispheres (Avidan et al., 2014; Song et al., 2015) or networks (Avidan et al., 2014; Zhao et al., 2018), we analyzed each region separately to detect abnormalities that may be specific to either the left or right hemisphere. Finally, we also performed whole-brain functional connectivity analyses for each seed region, making this the most comprehensive resting-state functional connectivity analysis in DP to date.

## 2 Materials and Methods

### 2.1 Participants

Thirty-four developmental prosopagnosics (DPs) were included in the study (six males; ages ranged from 20–70 years, *M* = 37.5, *SD* = 14.9). A total of 35 DPs were scanned (recruited from our database of DP participants from previous studies, references from other research laboratories, and through advertisements on the Massachusetts Bay Transportation Authority), but one was excluded due to excessive movement in the scanner (average framewise displacement > 0.5; Power et al., 2012).

Twenty-three neurotypical controls were included in the study (9 males; ages ranged from 18–63, *M* = 32.2, *SD* = 11.7). A total of 25 neurotypical controls were scanned, but one was excluded for scoring more than 2 *SD* below the control mean on the CFMT. A second was excluded due to excessive movement during the functional localizer. A third was excluded from just the functional connectivity analysis due to excessive movement during the resting-state scan. Thus, a total of 23 controls were included in the functional activation analysis and 22 controls were included in the functional connectivity analysis.

No significant differences between groups were found for age, *t*(55) = 1.75, *p* = .085, or gender, χ²(2) = 4.96, *p* = .084. All participants completed a self-report measure of face recognition (Prosopagnosia Index 20, PI20; Shah et al., 2015), a Famous Faces Memory Test (FFMT; Mishra et al., 2021), and the Cambridge Face Memory Test (CFMT; Duchaine & Nakayama, 2006).

#### 2.1.1 Diagnostic criteria for developmental prosopagnosia

Classic diagnostic cutoffs (i.e., self-reported difficulties along with > 2 *SD* below the control mean on both the CFMT and FFMT; DeGutis et al., 2023) have been shown to be overly strict and lead to the exclusion of roughly half of individuals reporting lifelong face recognition impairments (Bate et al., 2019; Burns et al., 2022; DeGutis & Campbell, 2024). The revised diagnostic criteria for inclusion in the DP group, therefore, consisted of self-reported difficulties and at least 1 *SD* below the control mean on *both* the CFMT and FFMT (for review, see DeGutis & Campbell, 2024; Sachdev et al., 2014). These revised criteria have been shown to identify 3.08% of the population as having DP (0.93% based on classic criteria and 2.15% based on the adapted DSM-5 criteria for mild deficits; see DeGutis et al., 2023). A single participant was unable to complete the Famous Face Memory Test due to technical difficulty, but was found to perform at prosopagnosic levels on several other assessments (e.g., *z* < -2 on the BFRT-c). Out of all 34 DPs, 27 met the threshold for the classic criteria (see Table 1). Scores on the PI20 ranged from 58–93. All DP participants scored within the normal range on the Leuven Perceptual Organization Screening Test (L-POST; Torfs et al., 2014).

**Table 1.** DP scores on three diagnostic measures.

	PI20	CFMT (raw)	CFMT (z-score)	FFMT (z-score)
DP1	88	34	-3.64	-4.42
DP2	88	37	-3.25	-3.75
DP3	75	39	-2.98	-3.91
DP4	89	38	-3.11	-4.31
DP5	93	35	-3.51	-2.20
DP6	80	38	-3.11	-5.13
DP7	75	34	-3.64	-3.45
DP8	86	35	-3.51	-3.36
DP9	80	42	-2.59	-2.77
DP10	58	40	-2.85	-2.06
DP11	77	47	-1.93	-2.53
DP12	86	44	-2.32	-4.24
DP13	63	37	-3.25	-5.03
DP14	89	37	-3.25	-5.86
DP15	87	47	-1.93	-3.75
DP16	78	33	-3.77	-1.11
DP17	80	42	-2.59	-4.47
DP18	91	33	-3.77	-3.78
DP19	80	39	-2.98	-2.80
DP20	85	43	-2.45	-4.61
DP21	92	45	-2.19	-3.94
DP22	88	44	-2.32	-4.47
DP23	82	44	-2.32	-5.03
DP24	86	52	-1.27	-2.35*
DP25	92	32	-3.91	-3.57
DP26	83	36	-3.38	-5.65
DP27	73	52	-1.27	-3.22
DP28	76	42	-2.59	-6.00
DP29	61	45	-2.19	-3.75
DP30	87	44	-2.32	-5.22
DP31	72	40	-2.85	-5.58
DP32	93	46	-2.06	-2.74
DP33	78	48	-1.79	-2.99
DP34	81	39	-2.98	-1.11

* denotes computerized Benton Facial Recognition Test score for participant who could not complete the Famous Face Memory Test.

PI20, Cambridge Face Memory Test (CFMT) and Famous Face Memory Test (FFMT).

#### 2.1.2 Exclusion criteria

The exclusion criteria for DP were the following: deficient L-POST scores, indicating poor low/mid-level vision; acquired prosopagnosia; a history of a significant neurological disorder, moderate to severe traumatic brain injury (TBI) or mild TBI in the last 6 months; musculoskeletal or sensory impairments that would interfere with performing computer tasks; lack of English proficiency; current psychiatric disorders or diagnosed social cognitive disorders such as autism; current dependence on alcohol or other substances. The same exclusion criteria applied for control participants, except they could not report everyday face recognition difficulties and were required to score 45 or above on the CFMT.

These sample sizes exceed those of the majority of prior studies that have reported DP differences in selectivity and/or functional connectivity (e.g., Gerlach et al., 2019; Rosenthal et al., 2017; Song et al., 2015). All participants provided informed consent before participation. The study was approved by the VA Boston Healthcare System and Harvard Medical School Institutional Ethical Review Board Committee, and all study tasks were completed at the Boston VA Medical Center or the Harvard Decision Science Lab.

### 2.2 Imaging data collection and MRI parameters

All scans were performed on a 3-Tesla Siemens Prisma using a 32-channel head coil. First, a high-resolution anatomical image was acquired using a T1-weighted magnetization prepared rapid gradient echo (MPRAGE) sequence using the following parameters: acquisition time (TA) 6:02 min, repetition time (TR) = 2530 ms, echo time (TE) = 3.35 ms, field of view (FOV) = 256 mm^2^, 177 slices, slice thickness = 1 mm, and flip angle = 7º. Functional images were collected using a T2*-weighted multiband accelerated echo-planar image (EPI) sequence using the following parameters: 72 slices, voxel size = 2 × 2 × 2 mm, flip angle = 52°, TR = 800 ms, TE = 37 ms, FOV = 208 mm x 208 mm^2^, and multiband accelerating factor = 8. Four hundred and eighty-eight volumes were collected for the rest scan, and 288 volumes for each of the four localizer scans.

### 2.3 Scan stimuli and experimental design

After the T1-weighted scan, a 6.5-min resting-state scan was performed. Participants were instructed to rest in the scanner with their eyes open. After the rest scan, functional data were acquired over four runs of a dynamic functional localizer of 234 s each. Using a blocked-design, each run contained three 18-s rest blocks at the start, middle, and end of the run, during which a sequence of six uniform color fields were presented for 3 s each. Between each of the rest blocks, five consecutive 18-s visual stimulus blocks were presented, each containing six 3-s video clips from one of five different stimulus categories: unfamiliar children’s faces, famous faces, scenes, bodies, and objects. Each run, therefore, contained two blocks for each category for a total of 10 visual stimulus blocks. Each block of the famous faces contained video clips of either Barack Obama or Donald Trump, readily identified by all participants. Clips of Obama and Trump were created from videos found on YouTube (see Fig. 4); all other stimuli were taken from Pitcher et al. (2011). The order of stimulus category blocks was palindromic (e.g., colour block, object, face, famous face-Obama, body, scene, colour block, scene, body, famous face-Trump, face, object, colour block), and counterbalanced across runs. Across the four runs, a total of 48 clips were presented for each stimulus category. There were 35 unique clips of bodies, 34 unique clips of faces (each showing one of 8 different identities), 33 unique clips of objects, 29 unique clips of scenes, 17 unique clips of Obama, and 16 unique clips of Trump. These were pseudorandomized across each of the runs, and no clip was repeated within the same run. Participants were instructed to passively view the video clips.

This study focused on face-selective ROIs using the unfamiliar faces > objects contrast, and localizer data for other categories (except for the contrast) were used for other purposes and not reported here.

### 2.4 MRI data preprocessing

Results included in this analysis come from preprocessing performed using fMRIPrep 1.3.0.post2 (Esteban et al., 2019), which is based on Nipype 1.1.8 (Gorgolewski et al., 2011).

The T1-weighted (T1w) image was corrected for intensity non-uniformity with N4BiasFieldCorrection (Tustison et al., 2010), distributed with ANTs 2.2.0 (Avants et al., 2008), and used as T1w-reference throughout the workflow. The T1w-reference was then skull-stripped using antsBrainExtraction.sh (ANTs 2.2.0), using OASIS30ANTs as target template. Brain surfaces were reconstructed using recon-all (FreeSurfer 6.0.1, Dale et al., 1999), and the brain mask estimated previously was refined with a custom variation of the method to reconcile ANTs-derived and FreeSurfer-derived segmentations of the cortical gray-matter of Mindboggle (Klein et al., 2017). Spatial normalization to the ICBM 152 Nonlinear Asymmetrical template version 2009c (Fonov et al., 2009) was performed through nonlinear registration with antsRegistration (ANTs 2.2.0), using brain-extracted versions of both T1w volume and template. Brain tissue segmentation of cerebrospinal fluid (CSF), white-matter (WM), and gray-matter was performed on the brain-extracted T1w using fast (FSL 5.0.9, Zhang et al., 2001).

The following preprocessing was performed for the functional images. First, a reference volume and its skull-stripped version were generated using a custom method from fMRIPrep. A deformation field to correct for susceptibility distortions was estimated based on two echo-planar imaging references with opposing phase-encoding directions, using 3dQwarp (Cox & Hyde, 1997, AFNI 20160207). Based on the estimated susceptibility distortion, an unwarped BOLD reference was calculated for a more accurate co-registration with the anatomical reference. The BOLD reference was then co-registered to the T1w reference using bbregister (FreeSurfer) which implements boundary-based registration (Greve & Fischl, 2009). Co-registration was configured with nine degrees of freedom to account for distortions remaining in the BOLD reference. Head-motion parameters with respect to the BOLD (transformation matrices, and six corresponding rotation and translation parameters) were estimated before any spatiotemporal filtering using mcflirt (FSL 5.0.9, Jenkinson et al., 2002). BOLD runs were slice-time corrected using 3dTshift from AFNI 20160207 (Cox & Hyde, 1997). The BOLD time-series were resampled to MNI152NLin2009cAsym standard space, generating a preprocessed BOLD run in MNI space. Several confounding time-series were calculated based on the preprocessed BOLD: framewise displacement (FD), derivative of RMS variance over voxels (DVARS), and three region-wise global signals. FD and DVARS are calculated for each functional run, both using their implementations in Nipype (following the definitions by Power et al. (2014)). Global signals were extracted within the CSF and the WM. Additionally, a set of physiological regressors were extracted to allow for component-based noise correction (CompCor, Behzadi et al., 2007). Principal components were estimated after high-pass filtering the preprocessed BOLD time-series (using a discrete cosine filter with 128s cut-off). A subcortical mask was obtained by heavily eroding the brain mask, which ensures it does not include cortical GM regions. Six aCompCor components are calculated within the intersection of the subcortical mask and the union of CSF and WM masks calculated in T1w space, after their projection to the native space of each functional run (using the inverse BOLD-to-T1w transformation). The head-motion estimates calculated in the correction step were also placed within the corresponding confounds file.

### 2.5 Face selectivity analysis

To compute unbiased estimates of face selectivity within-subject (Esterman et al., 2010; Kriegeskorte et al., 2009), we divided the four runs into localization and test runs to measure response of the voxels separately from the data used to localize them. In each of the leave-one-out combinations, three of the four localizer runs for a participant were used to localize the voxels that showed the strongest preference for faces (e.g., largest z-value for unfamiliar faces > objects contrast). To ensure that the magnitude of this response reflected face selectivity, only voxels showing a positive response for faces over objects from the three localizer runs were included. We then identified peak selectivity within the bilateral OFA, FFA, vATL, pSTS, and aSTS. These regions were defined using the maximum probability ROI map for faces from the brain activity atlas (www.brainactivityatlas.org; Zhen et al., 2015), with the exception of the vATL, which was defined by a custom anatomical mask created by combining regions from the Harvard-Oxford Atlas to provide coverage of the most inferior aspect of the temporal pole (temporal pole; inferior temporal gyrus, anterior and posterior divisions; temporal fusiform cortex, anterior division). We then used all voxels within a spherical ROI of 6 mm radius around each of these peaks to obtain ten individually localized ROIs. Selectivity was measured as the beta values of the unfamiliar faces > objects contrast in the left out run, averaged across the voxels in each of the spherical ROIs. The contrast beta values extracted from each run were then averaged to obtain each participant’s face selectivity for each ROI.

For each hemisphere and ROI region, we conducted linear regressions to examine the effect of group on face-selective activation across all ROIs, controlling for age and gender. As there were trending differences in age and gender between groups, each model included group, age, and gender as predictors. To control for multiple comparisons, FDR-correction was applied to *p*-values for the group predictor across all ten models. To compare our data with previous studies examining differences in the core and extended networks, we tested for group differences in the posterior (OFA, FFA, pSTS) and anterior (aSTS, vATL) regions using a 2 (region) x 2 (group) ANOVA with age and gender as covariates. We also tested for group differences in the ventral (OFA, FFA, vATL) and lateral (pSTS, aSTS) pathways using a 2 (pathway) x 2 (group) ANOVA with age and gender as covariates.

In addition to the face-selective response for unfamiliar faces, we quantified the magnitude of activation to familiar faces (i.e., Trump and Obama blocks from the category localizer) for each participant in the the face-selective regions defined using the unfamiliar faces > objects contrast from all four runs of the functional localizer. Linear models were used to determine the effect of group on activation to famous faces after controlling for age and gender. Lastly, because of the difference in sample sizes, a bootstrap analysis was conducted when equating for sample size. The results showed that statistical differences between the full DP sample and the control sample cannot be accounted for by having a larger number of DPs in the sample (see S1, Supplemental Materials).

### 2.6 Face-selective seed regions for functional connectivity

The face-selective seed regions for the resting-state functional connectivity analysis were individually defined for each participant using the unfamiliar faces > objects contrast from all four runs of the functional localizer. We then localized peak face selectivity within each of the face-selective areas described above to create ten spherical seed regions of 6 mm radius around each peak.

Time series data were extracted using the nilearn package (Pedregosa et al., 2011) for Python. We extracted the mean signal of interest from the seed regions as well as the brain-wide voxel-wise time series for the seed-to-voxel analysis. Time series data were detrended, standardized, and bandpass filtered (0.01–0.1 Hz). Six head-motion parameters and the first six aCompCor noise components (Behzadi et al., 2007) were regressed out during extraction. Correlation scores were Fisher *z* transformed before statistical analysis. Linear regression was used to determine the effect of group on overall network connectivity (i.e., averaged across all 45 pairwise correlations) and the mean functional connectivity for each seed region (i.e., averaged across all 9 pairwise correlations for each seed) after controlling for age and gender (FDR-corrected). We then conducted an exploratory analysis for each seed region where significant group differences were obtained to explore which pairwise correlations were different between groups. These are reported without FDR-correction. Finally, a bootstrap analysis was conducted to equate for sample size. Group differences in within-network connectivity were shown to be robust to sample size (see S1, Supplemental Materials).

### 2.7 Non-network functional connectivity

For each participant, we correlated the time series of each seed with all other brain voxels outside of the face network to obtain ten seed-to-voxel functional connectivity maps per participant. An inverse mask was used to exclude all voxels within the probabilistic face-selective regions and the vATL region. Fisher *z* transformation was applied to each of the seed-to-voxel correlation maps to normalize the correlation values before testing for group differences at the voxel-level to obtain ten difference maps with a *t*-score value representing the statistical difference in functional connectivity between groups. We then looked for voxel clusters where functional connectivity was significantly different at *p* < .01 (two-tailed) within each difference map with a cluster-correction of *p* < .05. After permuting group labels on the seed-to-voxel functional connectivity maps 10,000 times, the largest cluster observed on each permutation was used to create a distribution, and the threshold was set at the 95th percentile of the largest random clusters. To examine the possibility that group differences failed to survive the strict threshold of this statistical analysis, we also looked for clusters at a voxel-wise *p* < .01 and the corresponding cluster-correction at *p* < .05 (see S2, Supplemental Materials).

## 3 Results

### 3.1 Behavioral results

**Fig. 1.**
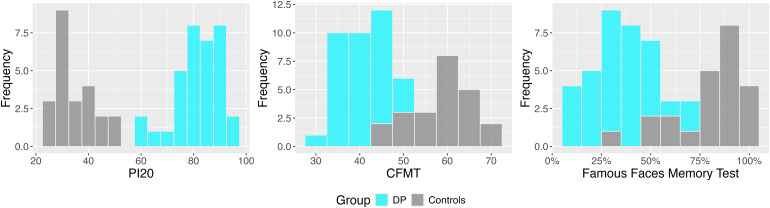
Developmental prosopagnosics were significantly impaired relative to controls on the two diagnostic memory tests and reported significantly more problems with everyday face recognition (PI20).

As predicted by the selection criteria, DP participants performed significantly worse than neurotypical controls (DP: *M* = 41, *SD* = 5; Controls: *M* = 59, *SD* = 7; *t*(55) = 11.4, *d* = 3.11, *p* < .001) on the Cambridge Face Memory Test (CFMT) and the Famous Face Memory Test (FFMT; DP: *M* = 35%, *SD* = 15%; Controls: *M* = 83%, *SD* = 16%; *t*(55) = 11.6, *d* = 3.17, *p* < .001). Based on the PI20 questionnaire, DPs also reported significantly more face recognition problems in their everyday lives (DP: *M* = 81, *SD* = 9; Controls: *M* = 34, *SD* = 7; *t*(55) = 20.8, *d* = 5.66, *p* < .001). Participant scores on these measures are shown in Figure 1.

### 3.2 Face selectivity

**Fig. 2.**
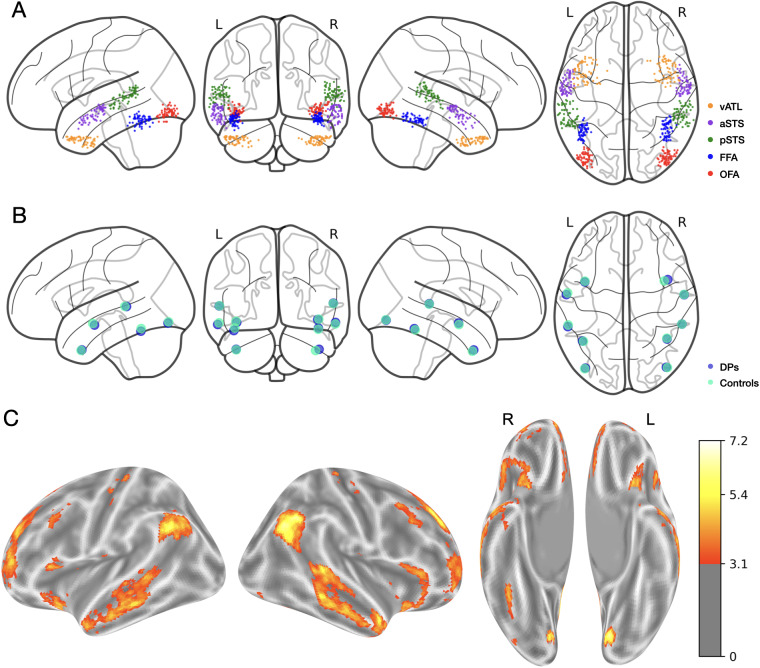
(A) Peak values for each participant’s individually-localized region-of-interest for the OFA (red), FFA (blue), vATL (orange), pSTS (green), and aSTS (purple). (B) Mean coordinates of individually-defined face-selective regions for developmental prosopagnosics (blue) and controls (turquoise) show a consistent spatial topography of the ROIs across DPs and controls. (C) The whole-brain significance maps (unfamiliar faces > objects, voxelwise *p* < .001, one-tailed, unadjusted) averaged across all participants. Only face-selective (positive z-values) values are shown.

Using the leave-one-run out procedure, we localized the bilateral OFA, FFA, pSTS, aSTS, and vATL in all participants for a total of 10 face-selective ROIs. The spatial distribution of the peak values is shown in Figure 2A. While there was substantial variability across individuals in the spatial location of each ROI (see Figs. S1, S2, and S3, Supplemental Materials), the average coordinates for each region were highly similar between groups (Fig. 2B). Whole-brain surface maps show the topography of the face-selective response, averaged across all participants (Fig. 2C). Mean MNI coordinates and subject-level contrast maps are provided in Supplemental Materials (Table S1).

**Fig. 3.**
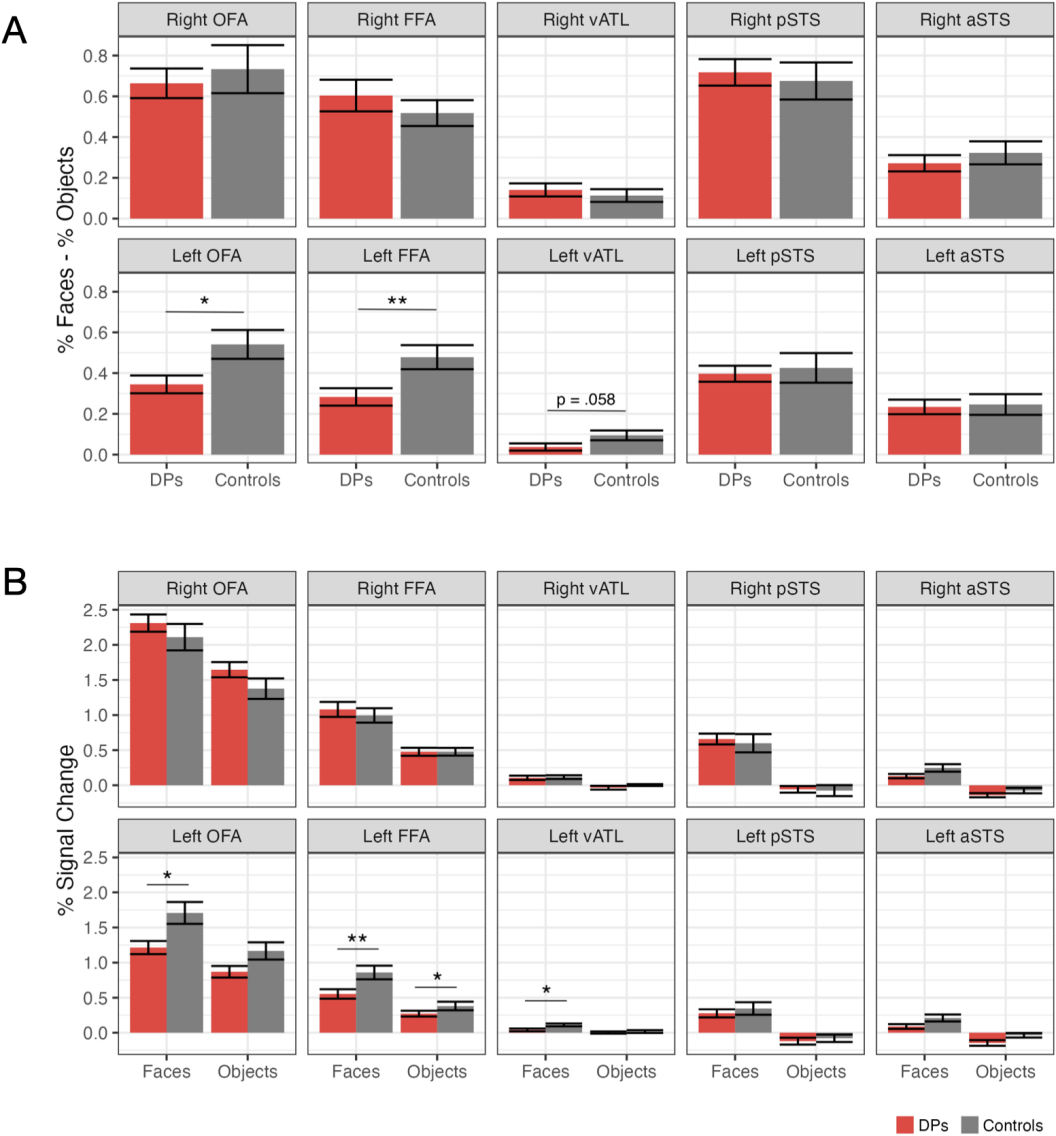
Face-selective activation differences (unfamiliar faces > objects), were observed between DPs and Controls in the left FFA (A) resulting from a reduced response to faces (B). A difference was observed in the left FFA (*p* = .004, *q* = 0.043) and the left OFA (*p* = .019, *q* = 0.10), although the reduction in the left OFA did not survive FDR-correction. No differences were observed in the right hemisphere regions. *p < .05, **p < .01 (unadjusted).

We conducted linear regressions to examine the effect of group on face-selective activation across all ROIs, controlling for age and gender. Each model included group, age, and gender as predictors^1^. A significant effect of group was observed in the left FFA (β = -0.40, unadjusted *p* = .004, *q* = 0.043), indicating reduced face-selective responses in the DP group compared to the controls. A similar effect was also observed in the left OFA (β = -0.34, unadjusted *p* = .019, *q* = 0.10), although this did not survive FDR-correction. A trend toward significance was also found in the left vATL (β = -0.28, unadjusted *p* = .058, *q* = 0.19). No other regions showed a significant group effect (see Fig. 3). We then examined the response to faces and objects separately for each of these three regions, again controlling for age and gender. For the left FFA, although DPs showed a significantly smaller response to objects (β = -0.29, unadjusted *p* = .011), the effect of group was even stronger and more robust for faces (β = -0.42, unadjusted *p* = .002). For both the left OFA and left vATL, the effect of group was significant only for faces (left OFA: β = -0.34, unadjusted *p* = .017; left vATL: β = -0.31, unadjusted *p* = .028). The results indicate that face-selective deficits in DP are more pronounced in the left hemisphere, particularly in the left FFA and left OFA.

Similar to previous studies examining differences between the posterior and anterior face network (e.g., Jiahui et al., 2018), we conducted a 2 (network) x 2 (group) ANOVA on the posterior (OFA, FFA, pSTS) and anterior (aSTS, vATL) regions. When controlling for age and gender as covariates, there was no effect of group on selectivity in the two networks (*p* = .72). Additionally, using the more recent distinction between the ventral and lateral processing pathways, a 2 (pathway) x 2 (group) ANOVA on ventral (OFA, FFA, and vATL) and the lateral (pSTS and aSTS) pathway ROIs, there was no effect of group on selectivity in the two pathways after controlling for age and gender (*p* = .28).

**Fig. 4.**
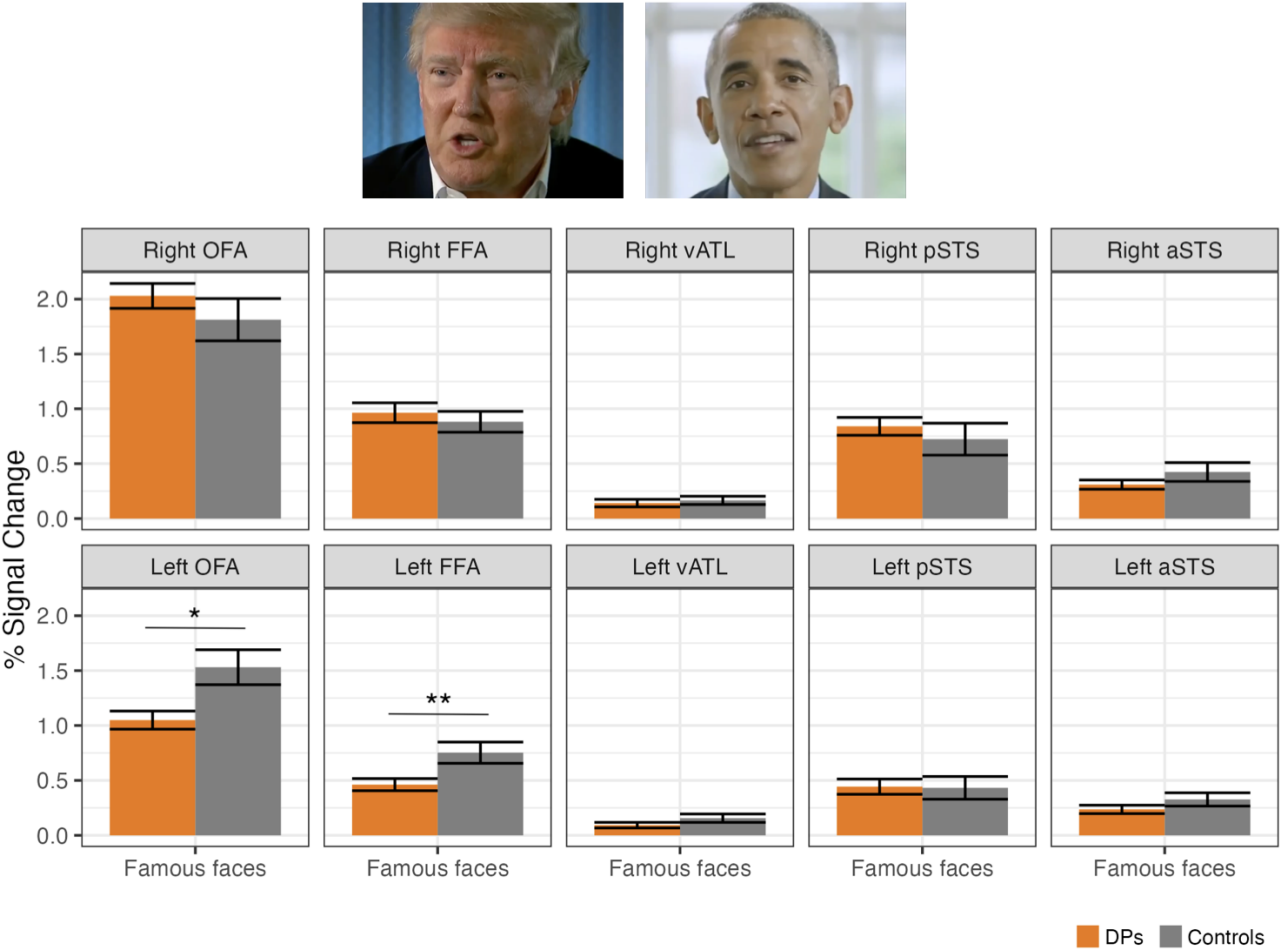
Percent signal change to famous faces (i.e., Obama and Trump blocks of the functional localizer) in the face-selective regions defined using the unfamiliar faces > objects contrast. Results show that DPs have a reduced response to familiar faces in the left FFA (*p* = .001, *q* = 0.01) and the left OFA (*p* = .018, *q* = 0.09), although the reduction in the left OFA did not survive FDR-correction. *p < .05, **p < .01 (unadjusted). (Trump: Screenshot from https://youtu.be/-GwG1_TkhQI, NBC News August 17, 2025; Obama: Screenshot from https://youtu.be/HwM9ROU5l4U, NBC News July 16, 2017).

To determine the generalizability of DP vs. control face activation differences, we then examined percent signal change to famous faces (i.e., Obama and Trump blocks of the functional localizer) in the face-selective regions defined using the unfamiliar faces > objects contrast from all four runs of the functional localizer (Fig. 4). We found the same pattern of deficits in DPs in response to these familiar faces: DPs had a significantly reduced response to faces in the left FFA (β = -0.44, unadjusted *p* = .001, *q* = 0.01) as well as the left OFA (β = -0.33, unadjusted *p* = .018, *q* = 0.09), although the left OFA did not survive FDR-correction.

For all selectivity analyses, the same pattern of results was obtained without the use of age and gender as covariates (see S3, Supplemental Materials).

### 3.3 Functional connectivity within the face network

**Fig. 5.**
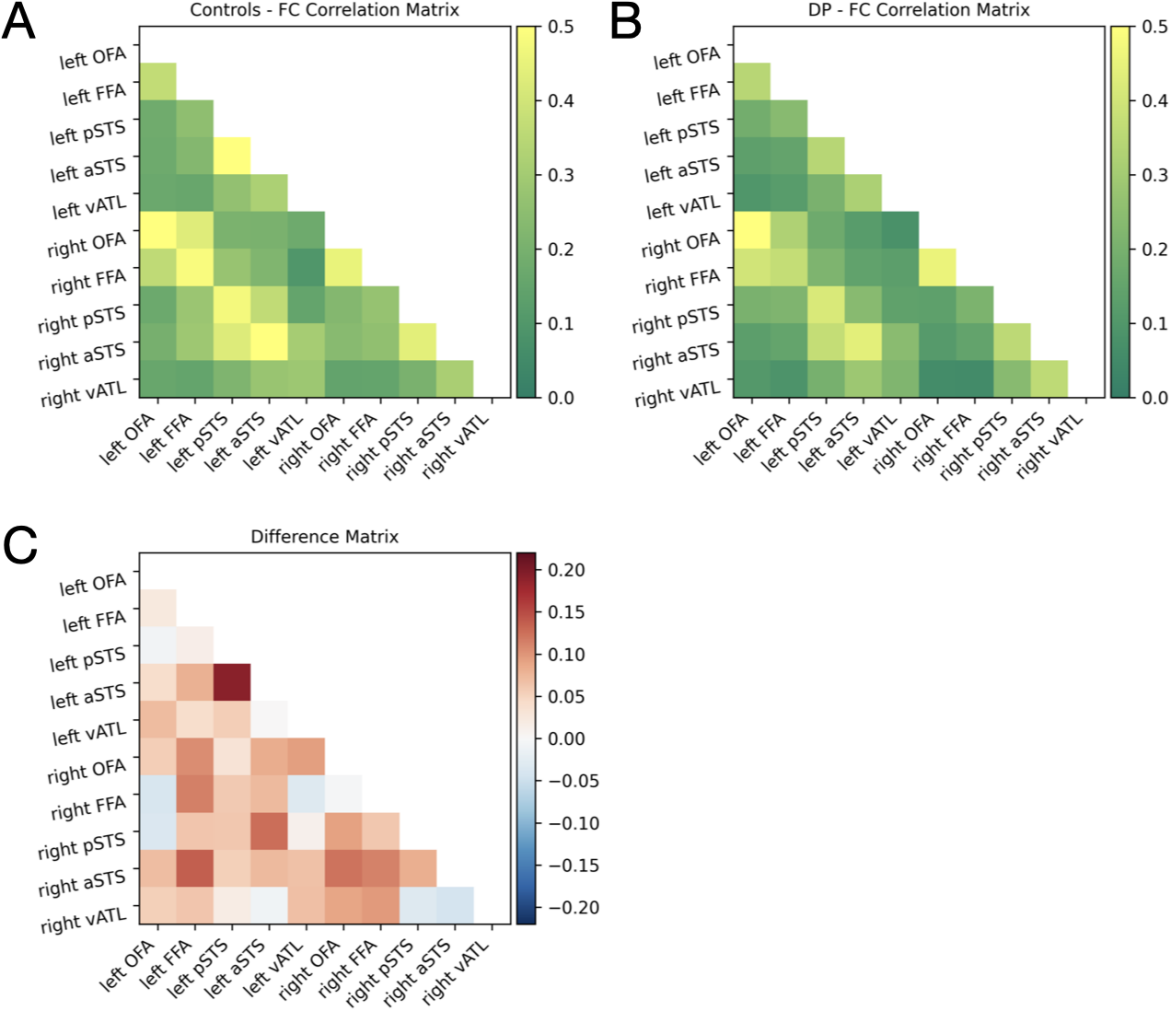
Average correlations within face-selective regions for control (A) and developmental prosopagnosic (DP; B) participants. The difference matrix (C) shows the difference in functional connectivity for each network edge (controls minus DP). In both groups, functional connectivity (FC) was strongest between bilateral regions. Overall, DPs showed reduced FC (*r* = .25) compared to controls (*r* = .31).

**Fig. 6.**
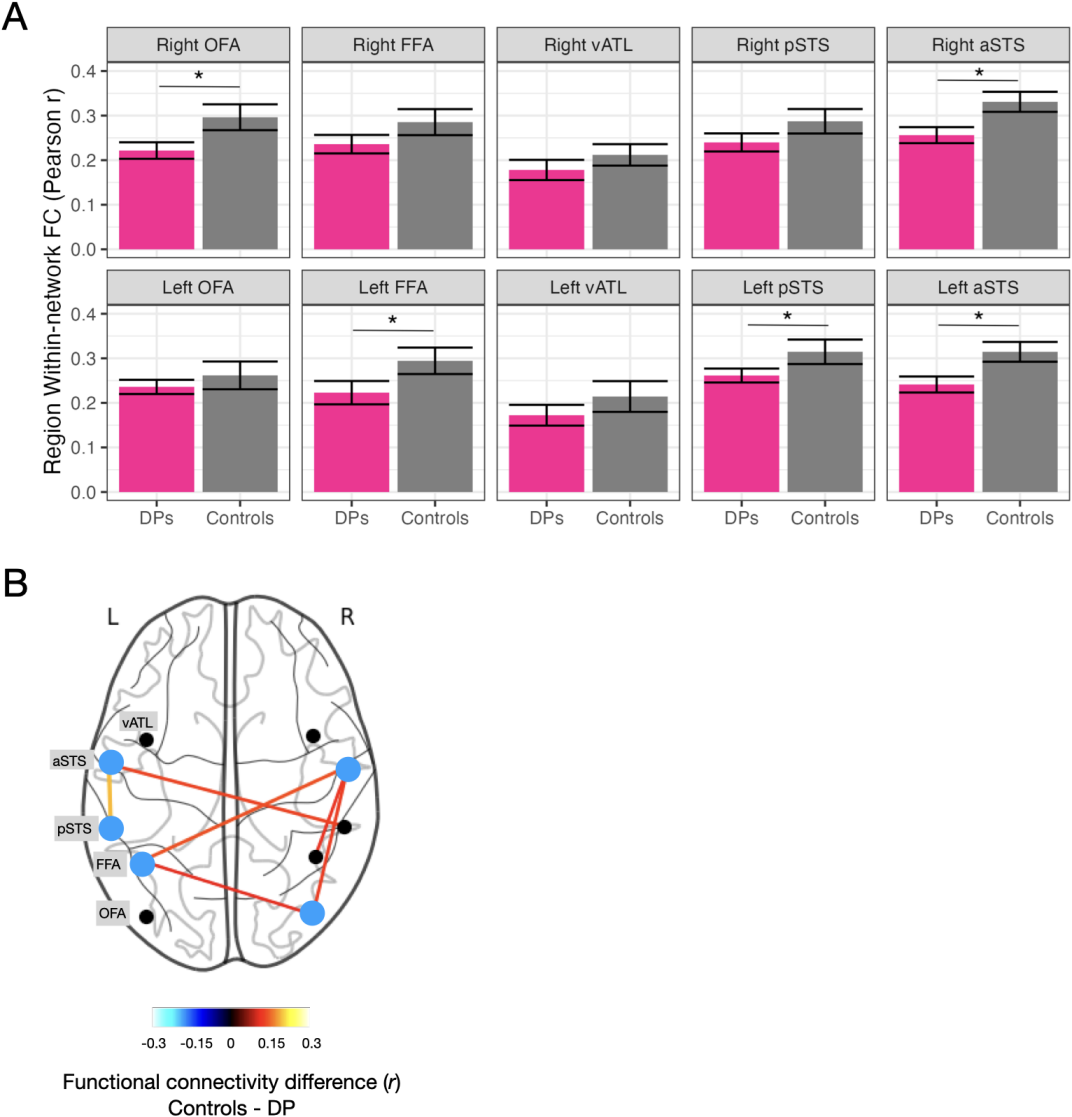
(A) Average network functional connectivity (FC) for each face network region was computed as the average of 9 pairwise correlations with all other face regions. DPs were found to have significantly reduced FC for the left FFA, right OFA, right aSTS, left aSTS, and left pSTS. *p < .05 (unadjusted). (B) Significant FC differences within the face network. Blue nodes represent a significant difference in average network FC. The color scale represents the difference in group-averaged FC (controls - DPs; Pearson *r*).

Using the resting-state data, we conducted linear regressions to examine the effect of group on resting-state functional connectivity within the face network, controlling for age and gender (Fig. 5). When averaging across all network connections (i.e., 45), there was a significant effect of group (β = -0.31, *p* = .035), indicating that overall network connectivity was reduced in DPs (*r* = 0.23) compared to controls (*r* = 0.28). To localize differences, we tested for group differences in the connectivity of each seed region for each hemisphere separately (i.e., the average of 9 pairwise correlations; see Fig. 6). Each model included group, age, and gender as predictors. A significant effect of group was observed in several regions, with DPs showing reduced functional connectivity compared to controls. In the left hemisphere, this was found for the left FFA (β = -0.31, unadjusted *p* = .032, *q* = 0.096), left pSTS (β = -0.31, unadjusted *p* = .039, *q* = 0.096), and left aSTS (β = -0.29, unadjusted *p* = .049, *q* = 0.098), although these did not survive FDR-correction. In the right hemisphere, the group effect was significant for the right OFA (β = -0.32, unadjusted *p* = .030, *q* = 0.096) and the right aSTS (β = -0.36, unadjusted *p* = .016, *q* = 0.096), although these also did not survive FDR-correction. The same pattern of results was obtained without the use of age and gender as covariates (see S3, Supplemental Materials).

We then examined the pairwise correlations for each of these regions to determine the specific edges of the network underlying DPs’ reduced overall connectivity (see Fig. 6). This showed that the right aSTS had the highest number of functional connectivity abnormalities with other regions of the face network (3/9 connections). See Table 2 for all group correlation values.

**Table 2.** Group comparisons of the average network connectivity values for each seed region.

Region	Network edge	Mean *r* Controls	Mean *r* DP	β	*p*
L OFA		0.26	0.24	-0.20	*ns*
**R OFA**		**0.30**	**0.22**	**-0.31**	**.030**
	L FFA	0.43	0.32	-0.29	.039
	R aSTS	0.23	0.11	-0.39	.007
**L FFA**		**0.29**	**0.22**	**-0.31**	**.032**
	R OFA	0.43	0.32	-0.29	.039
	R aSTS	0.29	0.15	-0.40	.007
R FFA		0.29	0.24	-0.21	*ns*
**L pSTS**		**0.31**	**0.26**	**-0.31**	**.039**
	L aSTS	0.53	0.34	-0.39	.005
R pSTS		0.29	0.24	-0.22	*ns*
**L aSTS**		**0.31**	**0.24**	**-0.29**	**.049**
	L pSTS	0.53	0.34	-0.39	.005
	R pSTS	0.36	0.24	-0.30	.038
**R aSTS**		**0.33**	**0.26**	**-0.36**	**0.016**
	L FFA	0.29	0.15	-0.40	.007
	R FFA	0.26	0.15	-0.34	.021
	R OFA	0.24	0.11	-0.39	.007
L vATL		0.21	0.17	-0.10	*ns*
R vATL		0.21	0.18	-0.10	*ns*
**Average within-network connectivity**		**0.28**	**0.23**	**-0.31**	**.035**

*Note:* The mean network connectivity values for each region are displayed as Pearson *r* values. Fisher’s transformation was applied to all correlation values before statistical analysis. Regions in bold showed significant DP vs. control differences. *p*-values are unadjusted. β – standardized beta.

As with our face selectivity analysis, a 2 (anterior/posterior) x 2 (control/DP) ANOVA did not find a group difference in seed connectivity in the anterior and posterior ROIs after controlling for age and gender (*p* = .47). A 2 (lateral/ventral) x 2 (control/DP) ANOVA also did not show a group difference in within-network connectivity of the two pathways after controlling for age and gender (*p* = .51). These results were replicated without the use of age and gender as covariates (see S3, Supplemental Materials).

### 3.4 Functional connectivity beyond the face network

To broaden the search for differences in functional connectivity to regions outside of the face network, seed-to-voxel functional connectivity maps were obtained for each participant and face region. We then tested for group differences in the whole-brain functional connectivity maps for each seed region separately. We found no clusters with different functional connectivity between the groups for any of the seed regions when thresholding at *p* < .001 (two-tailed *t*-test, cluster-corrected at *p* < .05). To examine the possibility that differences in functional connectivity failed to survive these strict thresholds, we also used a more liberal threshold of *p* < .01 (with a corresponding cluster correction of *p* < .05). However, we again found no evidence for functional connectivity differences beyond the areas surrounding the face-selective network seeds (see S2, Supplemental Materials).

### 3.5 Relationships between face selectivity and within-network connectivity

When examining ROI activation during passive face viewing, we found significant differences in the left FFA and, to a lesser extent, the left OFA. When examining functional connectivity (FC) during rest, we found two major differences: 1) the average within-network connectivity was reduced; 2) network connectivity of the left FFA, right OFA, left pSTS, and bilateral aSTS was reduced. To determine whether the task-driven activation differences were related to network connectivity observed during rest, we separately tested the correlation between activation of the left FFA and left OFA with each of the FC measures that were identified as abnormal. Since these regions were chosen based on observed group differences, we tested these correlations within each group separately. In DPs, we found no correlation between activation of the left FFA or left OFA and the key functional connectivity measures (all *p*s > .26). Similarly, we found no significant correlations within control participants (all *p*s > .12). The results, therefore, indicate that deficits in selective activation to faces during passive viewing are independent from the deficits in network FC during rest.

## 4 Discussion

This study provides a detailed analysis of face selectivity and functional connectivity (FC) abnormalities in developmental prosopagnosia for key face-selective regions in the ventral occipito-temporal cortex and lateral temporal cortex. When viewing faces, face-selective activation was reduced for DPs only in the left hemisphere in the left FFA and, to a lesser extent, the left OFA. During rest, functional connectivity averaged across the face network was reduced in DPs, but the largest reduction was found for the right aSTS region. Notably, we found no evidence for functional connectivity deficits extending beyond the areas surrounding the face-selective network regions. We discuss each of these findings in turn below.

First, when comparing unfamiliar face vs. object viewing, significant differences were confined to the left hemisphere, specifically in the left FFA and the left OFA, but with more reliable effects in the left FFA. No group-level differences were found in any right hemisphere region. Moreover, we observed the same pattern of results when examining the response to the familiar faces of Donald Trump and Barack Obama, indicating that abnormalities in the left FFA/OFA generalize to faces successfully recognized by our DP participants. While the observed response deficit of the left OFA in DPs is relatively novel (but see Avidan et al., 2014; Dinkelacker et al., 2011; Gerlach et al., 2019), the response deficit in the left FFA aligns with a broader pattern in the literature. Hemispheric effects have often been obscured due to the exclusion of left hemisphere face areas (DeGutis et al., 2007) or to collapsing across hemispheres (Avidan et al., 2014; Song et al., 2015) or across networks (Avidan et al., 2014; Zhao et al., 2018). However, a careful review of previous DP studies shows that reduced face selectivity is more consistently found in the left FFA (Avidan & Behrmann, 2009; Dinkelacker et al., 2011; Furl et al., 2011; Gerlach et al., 2019; Jiahui et al., 2018; Lohse et al., 2016) compared to the right (Avidan & Behrmann, 2009; Furl et al., 2011; Jiahui et al., 2018; Lohse et al., 2016).

Despite the recurring evidence of left hemisphere abnormalities in DP, these findings are often overlooked in accounts of its neural basis (but see Gerlach et al., 2019; Manippa et al., 2023). This is likely due to the dominant role of the right hemisphere in face processing models, along with a limited understanding of the functional role of the left hemisphere face-selective regions. In many ways, the left FFA appears to have similar functional properties as the right FFA. For both the left and right FFA, the magnitude (Elbich & Scherf, 2017; Furl et al., 2011; Huang et al., 2014; Weibert & Andrews, 2015; Yovel & Kanwisher, 2005) and spread (Elbich & Scherf, 2017; McGugin et al., 2016) of activation to faces has been shown to predict face recognition ability (and see S4, Supplemental Materials). The link to face memory has also been shown by Prince et al. (2009), who found that the BOLD response to faces in both the left and right FFA during study predicted subsequent recognition accuracy. Both the left and right FFA have also been implicated in facial identity processing using identity adaptation paradigms that show a reduced neural response to repeated images of the same identity that resets when identity changes (Ewbank & Andrews, 2008; Furl et al., 2011). Notably, DPs exhibit a reduced identity adaptation response in the left FFA but not the right FFA (Furl et al., 2011). However, functional asymmetries have also been found that suggest a larger role of the left FFA in local feature processing (Rossion et al., 2000), image-level analysis of face-like information (Meng et al., 2012), and perceptual learning (Bi et al., 2014). Although there is still no unifying theory, these findings suggest a more granular representation for face identity. For example, one study found that increased sensitivity to face views after perceptual training was associated with more consistent activation patterns elicited by a given image in the left but not the right FFA (Bi et al., 2014). Thus, it may be that the left FFA is important for establishing stable identity patterns to novel faces that are activated for subsequent encounters. Future studies should test whether these learning effects also extend to face identity processing.

Although mean functional connectivity averaged across all the face-selective regions was significantly reduced in DPs, we found that some regions were more affected than others. First, we found that DPs showed the largest reduction in functional connectivity for the right aSTS, and an exploratory analysis indicated that connectivity was especially reduced between the right aSTS and key regions for face perception: the right OFA and the FFA in both hemispheres. This may reflect abnormalities in a broader right hemisphere network for visual face recognition as indicated by behavioral (Gainotti, 2007, 2013), brain stimulation (Pisoni et al., 2020; Ranieri et al., 2015), and lesion studies (Glosser et al., 2003; Rice et al., 2018; Seidenberg et al., 2002) showing a greater role of the right anterior temporal lobe for face familiarity processing.

Large functional connectivity differences were also observed within the left temporal lobe itself, between the left pSTS and the left aSTS. This aligns with the recent finding that the familiarity response in the left STS is significantly diminished in DP compared to controls, and that this familiarity response strongly overlaps with face-selective regions of the STS (Noad et al., 2024). Moreover, DPs have been shown to have reduced gray matter volume in the bilateral STS relative to controls, suggesting a potential structural basis to the dysfunction (Garrido et al., 2009). Although the current design was not optimized for differentiating between perceptual and semantic face processing, prior work implicates the left lateral pathway in semantic processing (Lambon Ralph, 2014), object and face naming (Rice et al., 2018), and multimodal identity responses (Tsukiura et al., 2010; Y. Wang et al., 2017). Thus, the abnormally reduced connectivity between the left aSTS-pSTS observed in DPs likely contributes to their difficulty linking a face representation with identifying information, such as face-name associations (e.g., face-name deficits in DPs, Palsamudram et al., 2024). In combination with selectivity abnormalities, these findings highlight the need to better understand the functional role of the left hemisphere in face recognition.

These findings address a key limitation of previous DP studies that focus on the functional connectivity of the classic core network. Consequently, little was known about the functional connectivity within the “extended” network itself. For example, an initial analysis of a small group of DPs (N=7) examined resting-state functional connectivity between the core face network (created by averaging responses from the bilateral FFA, OFA, pSTS, and lateral occipital sulcus) and extended face network regions in the temporal lobe and found reduced connectivity between the core network and the right amygdala (Avidan et al., 2014). In a larger sample of 64 DPs, Zhao et al. (2018) examined resting-state functional connectivity between face-selective voxels in the core (i.e., OFA, FFA, and pSTS) and the extended network (i.e., voxels in all other face-selective regions identified by a face > object localizer). Their data showed that the right FFA was the only core network region where between-network connectivity was reduced in DPs, with reduced functional connectivity between the right FFA and large bilateral clusters along the STS. While our findings are consistent with this pattern, by using individually-localized regions for each participant (Zhao et al. used group averages), our approach provides a more detailed characterization of the connectivity abnormalities of the right aSTS to key areas for face perception as well as evidence for connectivity differences within the extended network itself (i.e., between the left pSTS and left aSTS).

Lastly, a key result from the functional connectivity analysis is that abnormalities in DP were effectively limited to the face network. This is consistent with the whole-brain analysis of healthy adults conducted by Wang et al. (2016) which showed that 95% of face-selective voxels had greater functional connectivity to other face-selective voxels than to voxels outside of the face-selective network. It has also been found that, in healthy controls, right OFA-FFA functional connectivity predicts both face recognition ability (using an old/new recognition task) and holistic face processing (using the face-inversion effect and the part-whole effect; Zhu et al., 2011). In contrast, Ramot et al. (2019) found that, in healthy adults, functional connectivity between face-selective seed regions and clusters dispersed throughout the frontal, occipital, parietal, and medial temporal lobes correlated with residualized CFMT scores (after controlling for car memory ability), but that functional connectivity within their face-selective seed regions did not. It was, therefore, proposed that functional connectivity to regions outside of the face network plays a greater role in face memory than within-network connectivity. According to this view, it would be expected that DP individuals with severe face memory deficits would show greater functional connectivity differences with regions outside the face network. Instead, we found little evidence for functional connectivity differences beyond the face network; even at a very liberal threshold, we found no functional connectivity differences beyond the areas surrounding the face-selective network seeds themselves. Overall, our results are more consistent with the view that face recognition ability is linked to functional connectivity of a large network of regions that primarily consists of areas that respond selectively to faces.

In conclusion, using a large sample of 34 DPs, we found that DPs had reduced face selectivity in the left fusiform face area (FFA), and to a lesser extent in the left occipital face area (OFA) as compared to controls. This highlights the association between the left FFA and lifelong face recognition problems, indicating a role that is often overlooked in current models of face processing. Additionally, resting-state functional connectivity disruptions in DPs were primarily observed within the cortical face network. Future research is needed to explore how these connectivity deficits relate to face learning and semantic processing of face identity that may lie at the core of developmental prosopagnosia.

## Supplementary Material

Supplementary Material

## Data Availability

The data analyzed in this study can be found at https://osf.io/cze82/

## Footnotes

1 Across models, age was not a significant predictor in any region (*p*s > .31). Gender showed a significant effect only in the right FFA (β = -0.30, unadjusted *p* = .035) and the left aSTS (β = 0.31, unadjusted *p* = .028).
