# Supplementary material for: Left hemisphere abnormalities in face-selective activation and functional connectivity in developmental prosopagnosia
Source: Imaging Neurosci (Camb). 2025 Nov 14;3:IMAG.a.971. doi: 10.1162/IMAG.a.971 (PMC12620909; doi:10.1162/IMAG.a.971)
Supplement: Supplementary Material [file IMAG.a.971_supp.pdf]

Supplemental Material

Table S1. Mean MNI coordinates for face-selective regions (all participants). Standard deviation in parentheses.

ROI	*x*	*y*	*z*
Right OFA	41 (± 5)	-80 (± 5)	-11 (± 4)
Left OFA	-40 (± 4)	-81 (± 5)	-13 (± 4)
Right FFA	41 (± 3)	-52 (± 7)	-20 (± 3)
Left FFA	-42 (± 4)	-54 (± 6)	-21 (± 4)
Right pSTS	56 (± 5)	-37 (± 7)	5 (± 6)
Left pSTS	-57 (± 6)	-39 (± 9)	3 (± 6)
Right aSTS	58 (± 4)	-8 (± 8)	-14 (± 6)
Left aSTS	-58 (± 4)	-7 (± 8)	-15 (± 6)
Right vATL	41 (± 7)	6 (± 9)	-41 (± 3)
Left vATL	-40 (± 8)	5 (± 9)	-41 (± 3)

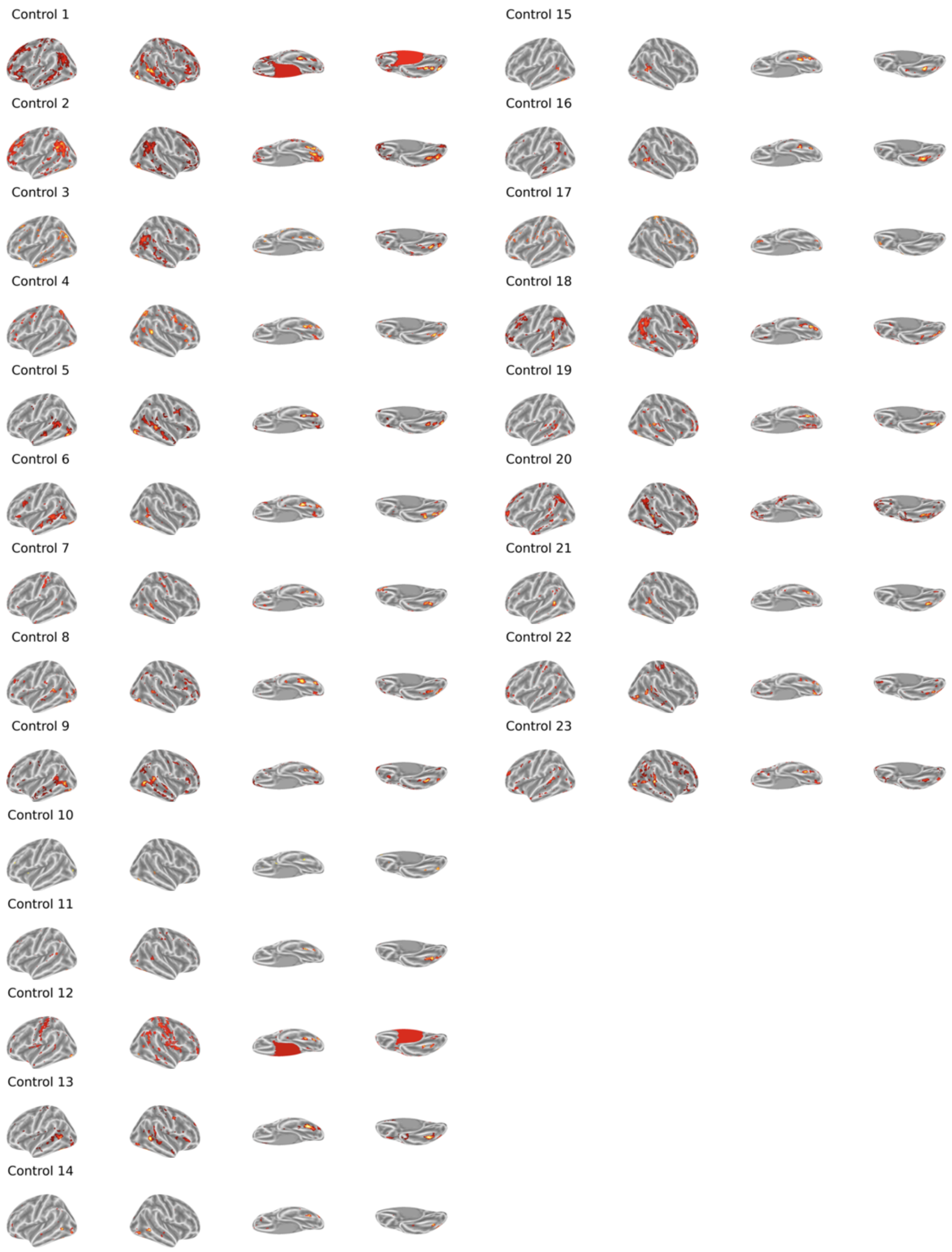

Figure S1. Whole-brain significance maps (unfamiliar faces > objects) for each control participant (N=23). Color reflects *z*-score, scaled for each participant, $z > 3.09$ (voxelwise $p < .001$, one-tailed, uncorrected).

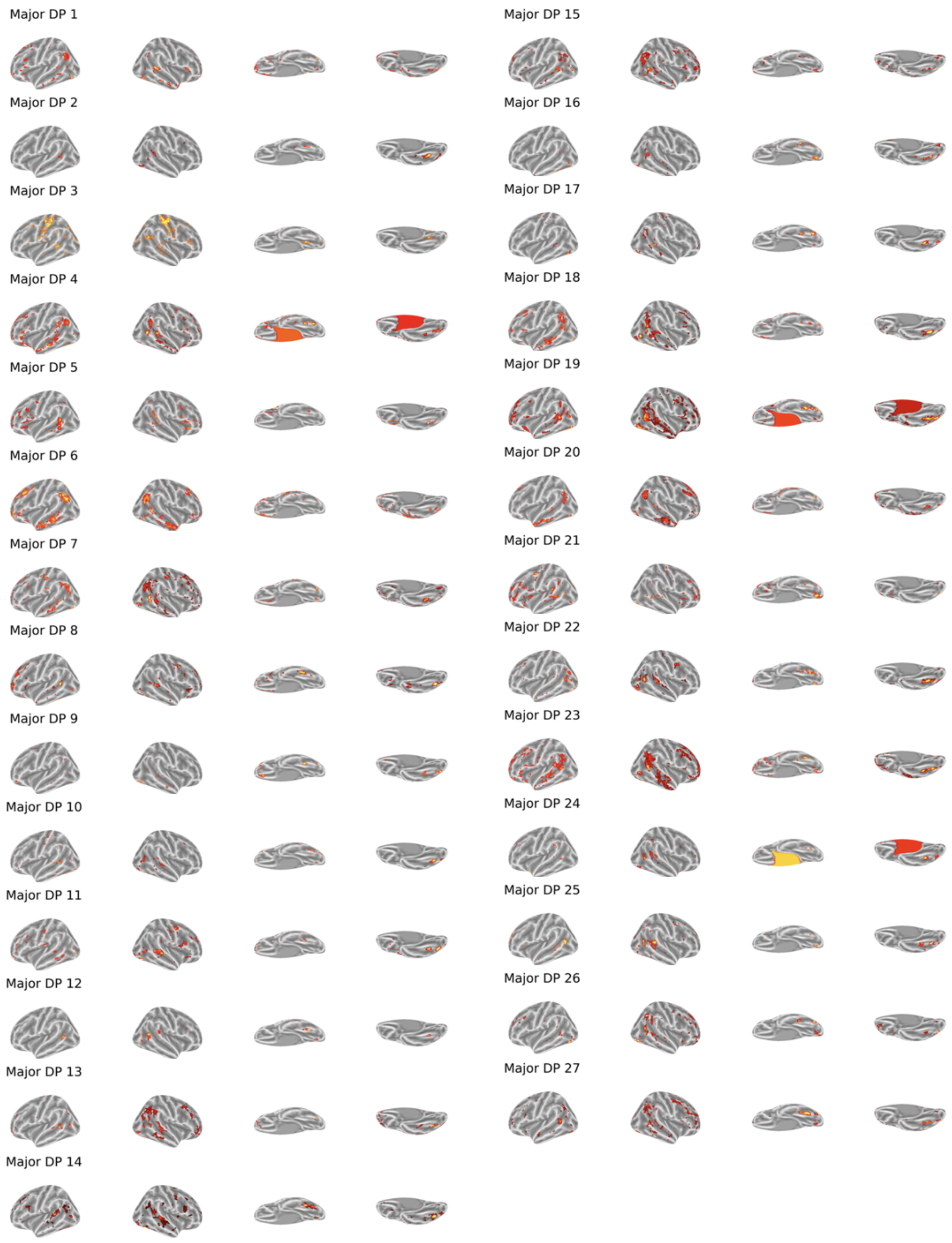

Figure S2. Whole-brain significance maps (unfamiliar faces > objects) for each DP participant classified as ‘major’ (N=27). Color reflects *z*-score, scaled for each participant, $z > 3.09$ (voxelwise $p < .001$, one-tailed, uncorrected).

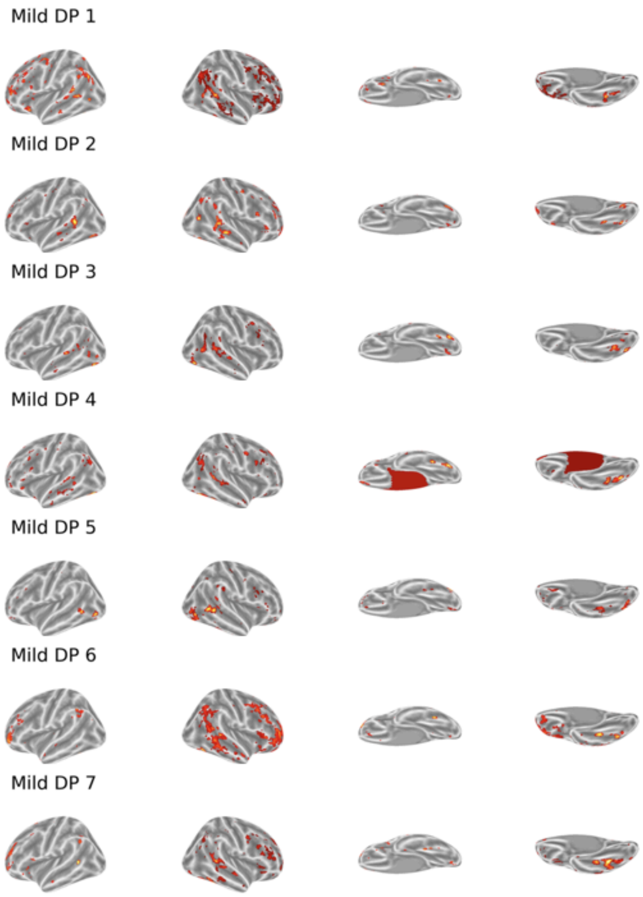

Figure S3. Whole-brain significance maps (unfamiliar faces > objects) for each DP participant classified as 'mild' (N=7). Color reflects *z*-score, scaled for each participant, $z > 3.09$ (voxelwise $p < .001$, one-tailed, uncorrected).

S1. Controlling for sample size differences using bootstrap analyses

Face selectivity

Analysis of the full sample of DP (N=34) and control participants (N=23) showed that DPs had reduced selectivity in the left FFA and left OFA. Given the unequal sample sizes, we then tested whether the observed group difference was robust to random subsampling of DPs to ensure that it wasn't driven by the larger size of the DP group. A bootstrapping procedure was carried out for the left FFA and left OFA separately. First, we drew 10,000 random subsamples of DPs (without replacement) that were equally sized to the control group (N = 23 for selectivity data) and computed mean selectivity for each. This generated an empirical distribution of mean selectivity values, from which we derived a 95% confidence interval (2.5th to 97.5th percentile). For both regions, the observed mean selectivity for controls falls above the 97.5th percentile of what would be expected from a random group of DPs. This indicates that, even when comparing equally sized groups, DPs consistently show lower selectivity in the left FFA and left OFA than controls.

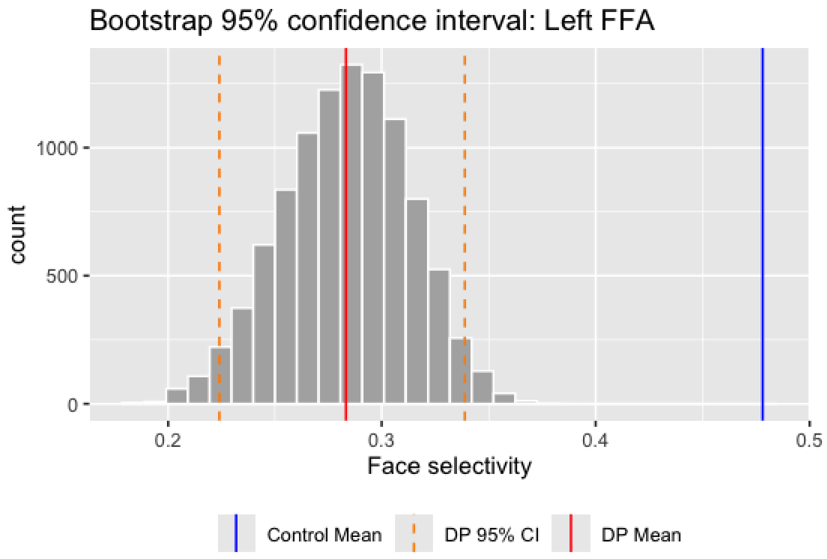

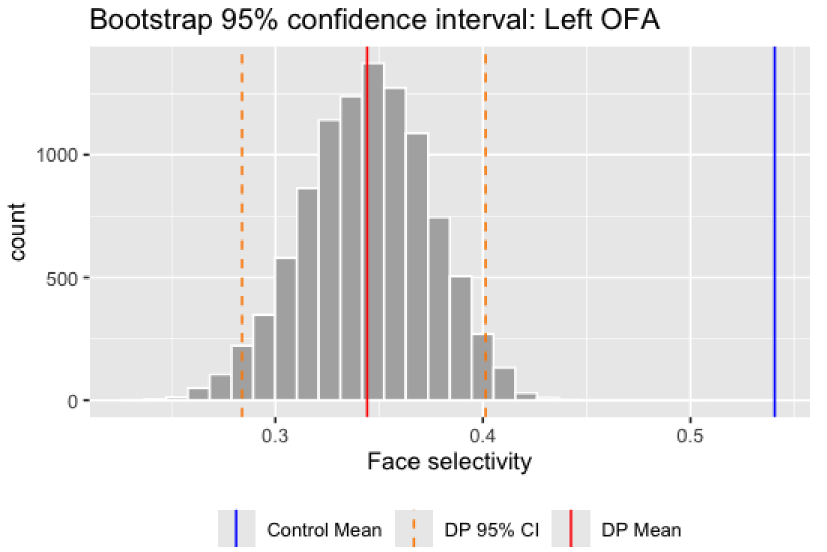

Functional connectivity

The full analysis showed that overall functional connectivity (FC) across all connections within the face network (i.e., 45 pairwise correlations) was significantly reduced in DPs compared to controls. Using a bootstrapping procedure, 95% confidence intervals

were obtained based on the distribution of mean FC from 10,000 DP subsamples that were equally sized to the control sample (N=22 for the FC analysis). The observed mean for controls lies above the 97.5th percentile of the bootstrap distribution, indicating that DPs consistently show reduced FC than controls when controlling for sample size.

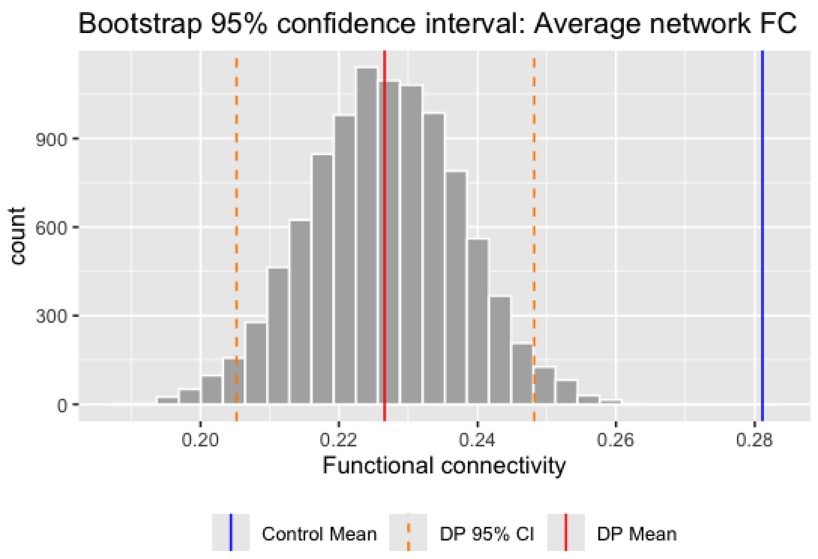

The full analysis also showed that there were several local regions that had reduced network FC in DPs compared to controls. These were the right OFA, left pSTS, and both the left and right aSTS regions. We therefore examined the mean FC of these regions in each of the 10,000 DP subsamples to compare it to the mean FC observed in the control sample. For each of these regions, the mean FC observed in the control sample exceeded the 95% confidence interval obtained from the bootstrap simulation. These simulations indicate that the reduced functional connectivity observed in the full DP sample is not an artifact of the DP group being larger.

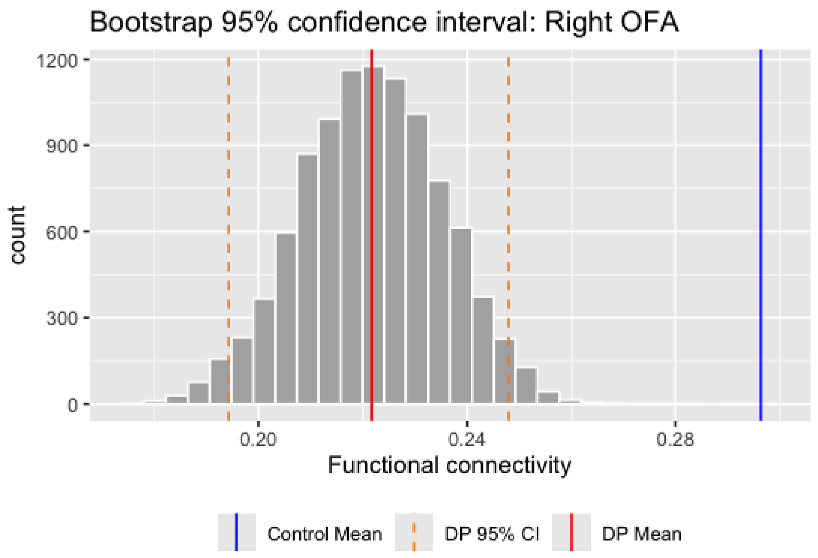

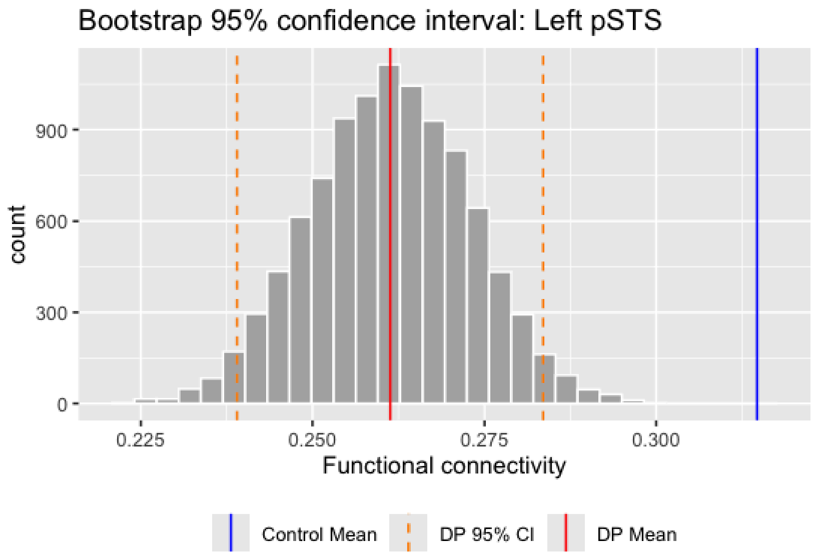
Bootstrap 95% confidence interval: Left pSTS
count
900
600
300
0
0.225
0.250
0.275
0.300
Functional connectivity
Control Mean
DP 95% CI
DP Mean

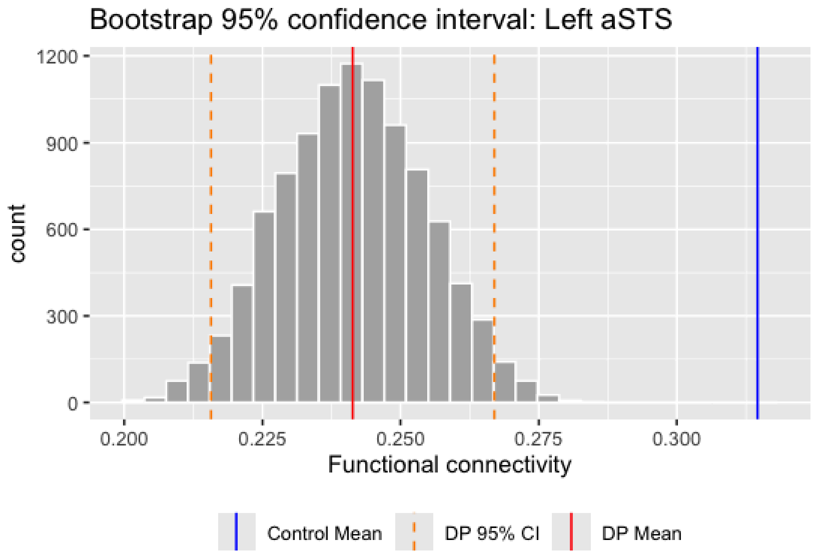
Bootstrap 95% confidence interval: Left aSTS
count
1200
900
600
300
0
0.200
0.225
0.250
0.275
0.300
Functional connectivity
Control Mean
DP 95% CI
DP Mean

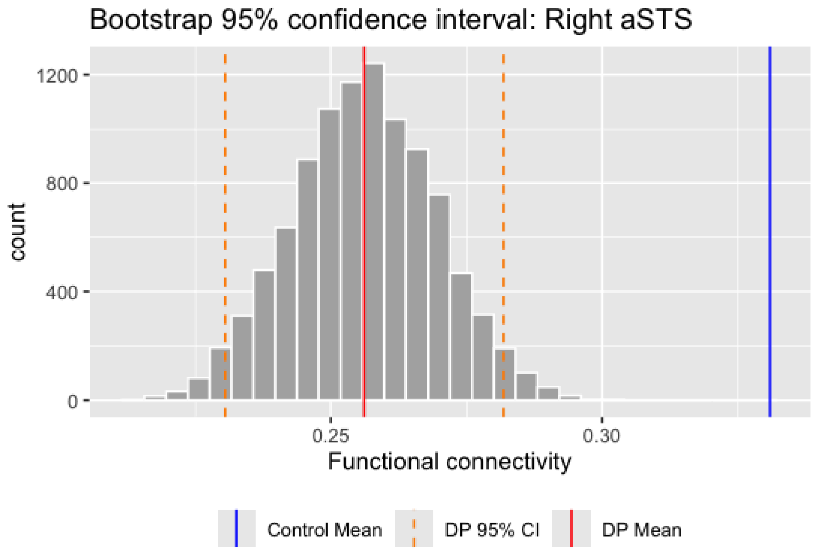
Bootstrap 95% confidence interval: Right aSTS
count
1200
800
400
0
0.25
0.30
Functional connectivity
Control Mean
DP 95% CI
DP Mean

S2. Whole-brain functional connectivity at a more liberal threshold

At a more liberal threshold (i.e., voxel-wise $p < .01$, cluster-corrected $p < .05$), we found some functional connectivity differences for both the left and right aSTS and the left vATL (Figure S4). Each of these regions were found to have a single cluster outside of the face network where functional connectivity was significantly reduced for DPs compared to controls. Specifically, DPs had reduced connectivity between each face-selective aSTS region and a large cluster of voxels adjacent to the contralateral STS. DPs also showed reduced connectivity between the left vATL and an expansive cluster of voxels running primarily along the posterior lateral occipital cortex.

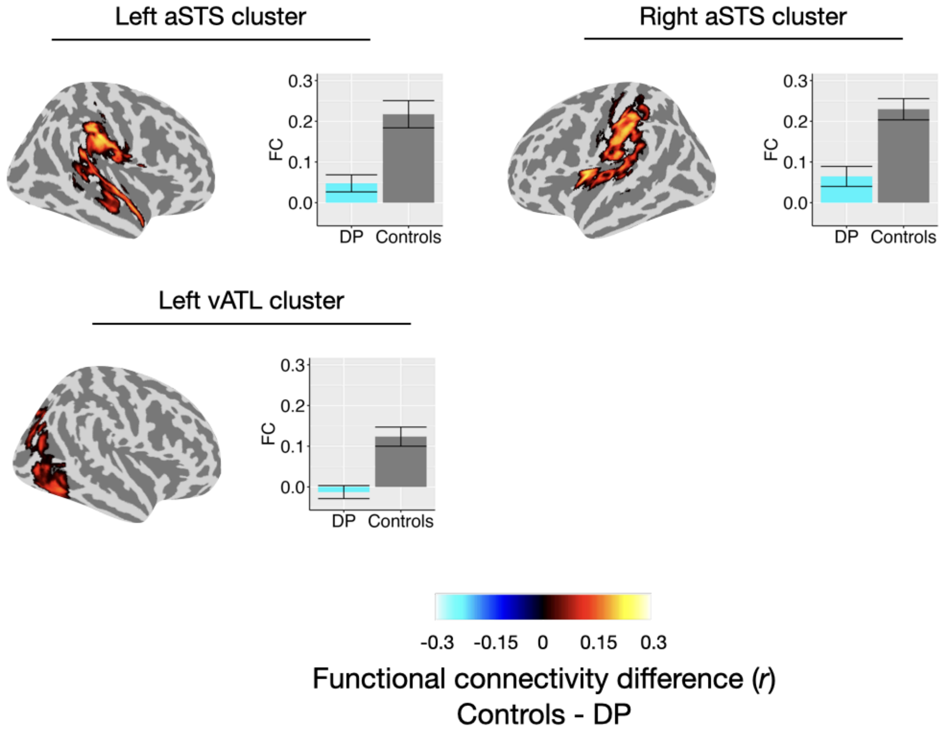

Figure S4. Seed-to-voxel functional connectivity showed that DPs also had reduced connectivity between certain network regions and clusters outside of the face network. Specifically, DPs were found to have reduced connectivity between each face-selective aSTS region and a large cluster of voxels adjacent to the contralateral STS. DPs also showed reduced connectivity between the left vATL and an expansive cluster of voxels running primarily along the posterior lateral occipital cortex. Bar graphs for each cluster show the group-averaged seed-to-voxel functional connectivity (Pearson *r*) across all cluster voxels.

S3. Statistical analyses without controlling for age and gender

Face selectivity

In the right hemisphere, there were no significant differences in face selectivity for DPs compared to controls. In the left hemisphere, DPs had significantly reduced face selectivity in the left FFA [$t(55) = 2.74$, $d = 0.73$, Bonferroni-Holm $p = .041$]. This was driven by a reduced response to faces [$t(55) = 2.67$, Bonferroni-Holm $p = .040$], and not a difference in response to objects ($p = .387$). DPs also exhibited significantly reduced face-selectivity in the left OFA ($p = .0157$), although this did not survive the Bonferroni-Holm correction, $t(55) = 2.49$, $d = 0.67$, Bonferroni-Holm $p = .063$. This was associated with a reduced response to faces [$t(55) = 2.88$, Bonferroni-Holm p = .028] but not objects ($p = .21$). We also found a reduced face-selective response for DPs in the left vATL that was marginally significant, $t(55) = 1.96$, $d = 0.53$, unadjusted $p = .055$.

A 2 (anterior/posterior) x 2 (control/DP) ANOVA on the posterior (OFA, FFA, pSTS) and anterior (aSTS, vATL) regions across hemispheres did not find a group difference in face selectivity in the two networks [$F(1, 55) = 0.34$, $p = .56$]. Additionally, using the more recent distinction between the ventral and lateral processing pathways, a 2 (ventral/lateral) x 2 (control/DP) ANOVA on ventral (OFA, FFA, and vATL) and the lateral (pSTS and aSTS) stream ROIs across hemispheres did not find a group difference in face selectivity of the two streams [$F(1, 55) = 1.33$, $p = .26$].

We then examined percent signal change to famous faces (i.e., Obama and Trump blocks of the functional localizer) in the face-selective regions defined using the unfamiliar faces > objects contrast from all four runs of the functional localizer. We found the same pattern of deficits in DPs in response to these familiar faces: DPs had a significantly reduced response to faces in the left FFA and as well as the left OFA [left FFA: $t(55) = 2.80$, $d = 0.76$, Bonferroni-Holm $p = .028$; left OFA: $t(55) = 2.93$, $d = 0.79$, Bonferroni-Holm $p = .025$].

Functional connectivity

When averaging across all network connections (i.e., 45), overall network connectivity was reduced in DPs ($r = 0.23$) compared to controls ($r = 0.28$), $t(54) = 2.17$, $d = 0.58$, $p = .035$. To localize differences, we tested for group differences in the connectivity of each seed region for each hemisphere separately (i.e., the average of 9 pairwise correlations). In the right hemisphere, DPs were found to have reduced network connectivity for the right OFA [t(54) = 2.30, $d = 0.61$, $p = .025$] and the right aSTS [$t(54) = 2.47$, $d = 0.67$, $p = .017$], although these did not survive Bonferroni-Holm correction. In the left hemisphere, DPs showed reduced network connectivity for the left pSTS [$t(54) = 2.02$, $d = 0.53$, $p = .048$] and left aSTS [$t(54) = 2.46$, $d = 0.67$, $p = .017$], yet these differences also did not survive a Bonferroni-Holm correction. Connectivity differences were also marginally significant for the left FFA [$t(54) =1.77$, $d = 0.49$, $p = .082$].

We then examined the pairwise correlations for each of these regions to determine the specific edges of the network underlying DPs' reduced overall connectivity. This showed that the right aSTS had the highest number of functional connectivity abnormalities with other regions of the face network (3/9 connections).

As with our face selectivity analysis, a 2 (anterior/posterior) x 2 (control/DP) ANOVA did not find a group difference in seed connectivity in the anterior and posterior ROIs [group x region interaction: $F(1, 54) = 0.02$, $p = .88$]. A 2 (lateral/ventral) x 2 (control/DP) ANOVA on the lateral (pSTS and aSTS) and ventral (OFA, FFA, and vATL) stream ROIs also did not show a group difference in within-network connectivity of the two streams [$F(1, 54) = 0.79$, $p = .38$].

S4. Brain-behaviour associations

Brain-behaviour relationships were examined by correlating each of the two face recognition measures (i.e., the Cambridge Face Memory Test and the Famous Faces Memory Test) with face-selective activation as well as functional connectivity for each of the face-selective regions. However, these relationships are different to interpret meaningfully for two reasons. First, because CFMT scores are one of the main diagnostic measures used to classify individuals as DP, any correlation is likely to be inflated by categorical group differences. Because the selection criteria creates a highly non-normal distribution of CFMT scores across all participants, any correlation may largely reflect between-group differences rather than a continuous, monotonic relationship across individuals. This statistical artifact occurs when group membership drives both the predictor and outcome variable, leading to spurious correlations and/or exaggerated correlation coefficients. Second, because individuals are included in the DP group only if their CFMT scores are lower than controls, the CFMT scores for DPs are truncated and lack variability. This restriction of range attenuates any within-group correlation that might exist in the DP group, further complicating the interpretation. Although we report on these correlations for reader interest, they should be interpreted with appropriate caution.

Face selectivity and CFMT

For each region-of-interest, we correlated face selectivity (unfamiliar faces > objects) with Cambridge Face Memory Test (CFMT) scores. Across all participants, we found significant correlations between face-selective activation and CFMT scores in the left OFA ($r = .42$, $p = .001$) and the left FFA ($r = .41$, $p = .002$). When each group was examined separately, the correlation in the left OFA was not significant for either group (DP: $r = .31$, $p = .08$; Controls: $r = .28$, $p = .20$). Within controls, a significant correlation was found for the left FFA ($r = .51$, $p = .012$) but not for DPs ($r = -.05$, $p = .77$). Additionally, a significant correlation between face selective responses in the right pSTS and CFMT scores was found for DPs ($r = .38$, $p = .028$) but not controls ($r = -.13$, $p = .56$). No other significant correlations were found.

Face selectivity and Famous Face Recognition

Across all participants, we found significant correlations between face-selective activation and Famous Face Memory Test (FFMT) scores in the left OFA ($r = .41$, $p = .002$) and the left FFA ($r = .39$, $p = .003$). When each group was examined separately, a significant correlation was found for the left OFA in controls ($r = .45$, $p = .031$) but not for DPs ($r = -.02$, $p = .90$). The correlation in the left FFA was not significant for either group (DP: $r = .20$, $p = .27$; Controls: $r = .20$, $p = .37$).

Functional connectivity and CFMT

For each participant, we computed the average functional connectivity (FC) for each seed region (i.e., the average of 9 pairwise correlations) and correlated these values with CFMT scores. Across all participants, significant correlations were observed between CFMT scores and functional connectivity of both the right ($r = .31$, $p = .019$) and left ($r = .26$, $p = .049$) aSTS regions. When examining each group separately, no significant correlations were found.

Functional connectivity and Famous Face Recognition

Across all participants, we found no significant correlations between functional connectivity of any of the seed regions and FFMT scores. There were also no significant correlations within either group.
